# Serum 25-hydroxyvitamin D concentration in childhood and risk of islet autoimmunity and type 1 diabetes: the TRIGR nested case–control ancillary study

**DOI:** 10.1007/s00125-019-05077-4

**Published:** 2020-01-07

**Authors:** Maija E. Miettinen, Sari Niinistö, Iris Erlund, David Cuthbertson, Anita M. Nucci, Jarno Honkanen, Outi Vaarala, Heikki Hyöty, Jeffrey P. Krischer, Mikael Knip, Suvi M. Virtanen, TRIGR Investigators

**Affiliations:** 1 Department of Public Health Solutions, National Institute for Health and Welfare, PO Box 30, 00271 Helsinki, Finland; 2 Department of Government Services, National Institute for Health and Welfare, Helsinki, Finland; 3 Health Informatics Institute, Morsani College of Medicine, University of South Florida, Tampa, FL USA; 4 Department of Nutrition, Georgia State University, Atlanta, GA USA; 5 Scientific Laboratory, Clinicum, University of Helsinki, Helsinki, Finland; 6 Faculty of Medicine and Health Technology, Tampere University, Tampere, Finland; 7 Fimlab Laboratories, Pirkanmaa Hospital District, Tampere, Finland; 8 Children’s Hospital, University of Helsinki and Helsinki University Hospital, Helsinki, Finland; 9 Folkhälsan Research Center, Helsinki, Finland; 10 Research Program for Clinical and Molecular Metabolism, University of Helsinki, Helsinki, Finland; 11 Department of Pediatrics, Tampere University Hospital, Tampere, Finland; 12 Faculty of Social Sciences, Health Sciences, University of Tampere, Tampere, Finland; 13 Research, Development and Innovation Center, Tampere University Hospital, Tampere, Finland; 14 Center for Child Health Research, Tampere University and Tampere University Hospital, Tampere, Finland

**Keywords:** 25-Hydroxyvitamin D, Islet autoimmunity, Type 1 diabetes, Vitamin D

## Abstract

**Aims/hypothesis:**

Our aim was to study the association between serum 25-hydroxyvitamin D (25OHD) concentration and islet autoimmunity and type 1 diabetes in children with an increased genetic risk of type 1 diabetes.

**Methods:**

Serum samples for 25OHD measurements were obtained in the Trial to Reduce IDDM in the Genetically at Risk (TRIGR) ancillary study (Divia) from children in 15 countries. Case children (*n* = 244) were defined as having positivity for at least two out of four diabetes-associated autoantibodies measured at any one sample. For each case child, two control children were selected matched for country and date of birth (±1 year) (*n* = 488). Of the case children, 144 developed type 1 diabetes. Serum 25OHD was measured repeatedly in infancy and childhood and was compared according to age at the first seroconversion (at 6, 12 and 18 months prior to and at seroconversion) and calendar age (0, 6, 12 and 18 months).

**Results:**

In children with islet autoimmunity, mean serum 25OHD concentration was lower 18 months prior to the age of first seroconversion of the case children compared with the control children (57.7 vs 64.8 nmol/l, *p* = 0.007). In children with type 1 diabetes (*n* = 144), mean serum 25OHD concentration was lower 18 months prior to the age of the first seroconversion (58.0 vs 65.0 nmol/l, *p* = 0.018) and at the calendar age of 12 months (70.1 vs 75.9 nmol/l, *p* = 0.031) than in their control counterparts. Analyses were adjusted for month of sample collection, human leucocyte antigen genotype, maternal type 1 diabetes and sex.

**Conclusions/interpretation:**

The results suggest that early postnatal vitamin D may confer protection against the development of type 1 diabetes.

**Trial registration:**

ClinicalTrials.gov NCT00179777

**Electronic supplementary material:**

The online version of this article (10.1007/s00125-019-05077-4) contains contains peer-reviewed but unedited supplementary material, which is available to authorised users.

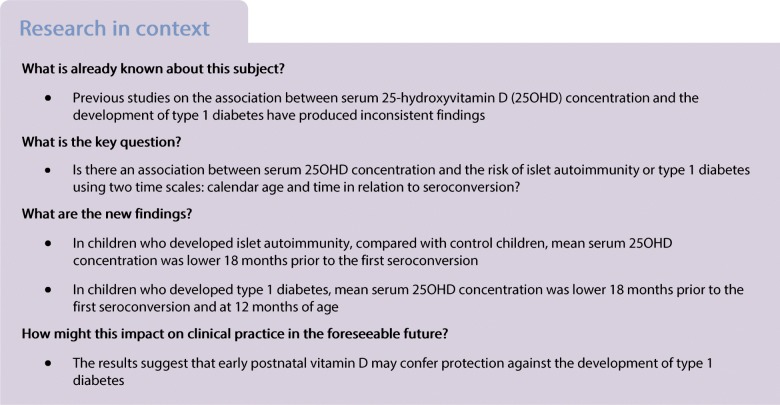



## Introduction

Type 1 diabetes is an immune-mediated disease in which insulin-producing beta cells in the pancreas are damaged. The preclinical phase of the disease can last from months to even decades [1] and is characterised by the presence of diabetes-associated autoantibodies, the most important of which are islet cell autoantibodies (ICAs), insulin autoantibodies (IAAs), insulinoma-associated antigen-2 autoantibodies (IA-2As), GAD autoantibodies (GADAs) and zinc transporter-8 autoantibodies (ZnT8As).

Vitamin D deficiency during the fetal period, infancy or childhood is one of the environmental factors implicated in the aetiology of type 1 diabetes, because of the active role of vitamin D in regulating the immune system. In previous studies investigating the association between vitamin D and type 1 diabetes, emphasis has been on either serum 25-hydroxyvitamin D (25OHD) concentration (regarded as a marker of vitamin D stores) arising from dietary and supplemental vitamin D intake or on vitamin D metabolism-related genetic factors.

Large birth cohort studies that have invited participants based on an increased genetic risk for type 1 diabetes have analysed the association between serum 25OHD concentration and type 1 diabetes. The Finnish Diabetes Prediction and Prevention (DIPP) study [2,3] and the Diabetes Auto Immunity Study in the Young (DAISY) [4] did not find any association between serum 25OHD concentration and risk of islet autoimmunity or type 1 diabetes at birth or during childhood, whereas the Environmental Determinants of Diabetes in the Young (TEDDY) study reported an association between low serum 25OHD concentration during childhood and increased risk of islet autoimmunity [5]. A Norwegian study found an association between maternal low serum 25OHD concentrations and increased risk for type 1 diabetes in the offspring [6], whereas in Finland such an association was not seen in a similar study setting [7]. Two other large studies from Norway and Denmark did not find an association between type 1 diabetes and serum 25OHD concentration at multiple time points from the first trimester of pregnancy until birth, or between type 1 diabetes and neonatal 25OHD concentration measured from dried blood spots [8,9].

Studies that have evaluated intake of vitamin D from the diet and/or supplements in relation to the risk of islet autoimmunity and type 1 diabetes, have produced mixed results [10]. In addition to the dietary intake, several factors modify serum 25OHD concentration including genetic factors and the amount of sunlight. Therefore, it may be challenging to determine whether higher intake of vitamin D from the diet or supplements has resulted in an improved vitamin D status, especially if the amount of recommended supplementation has been low. In Finland, a relatively high amount of daily vitamin D supplementation (50 μg) was recommended to infants during the first year of life in the 1960s. In a prospective birth cohort study, the risk of type 1 diabetes was found to be reduced by 80% in those receiving regular vitamin D supplementation, but the number of diabetes cases was low [11].

Vitamin D metabolism-related genetic factors have shown associations with type 1 diabetes but in genome-wide association studies only one SNP has reached statistical significance (rs10877012 in the *CYP27B1* gene) [12]. In the TEDDY study, it was noticed that the association between serum 25OHD concentration and islet autoimmunity was stronger among carriers of a genotype of a certain SNP (rs7975232) in *VDR*, the vitamin D receptor (VDR) gene [5]. Also in a recent Norwegian study, the association between serum 25OHD concentration at birth and risk of type 1 diabetes depended on the *VDR* genotype (rs11568820) [13]. *VDR* is known to regulate expression of hundreds of genes of which several are related to the function of the immune system [14].

The disease process of type 1 diabetes may start early in infancy with mainly unknown factors causing changes in the immune system even before the islet autoantibodies can be detected [15]. Based on the existing evidence, the possible role of vitamin D in the disease process of type 1 diabetes or importance of timing of inadequate vitamin D supply is not clear and may depend on the population and vitamin D status. Our aim was to investigate the possible role of serum 25OHD concentration during early childhood as a predictor of later risk of developing islet autoimmunity or clinical type 1 diabetes, both according to the age in relation to the first seroconversion and calendar age.

## Methods

### Study population

The current study is based on samples collected in the Trial to Reduce IDDM in the Genetically at Risk (TRIGR) cohort (ClinicalTrials.gov registration no. NCT00179777). The TRIGR study is an international double-blind randomised clinical trial of 2159 infants with HLA-conferred disease susceptibility and a first-degree relative with type 1 diabetes recruited between 2002 and 2007 in 15 countries [16]. The inclusion criteria of the TRIGR study included both certain HLA-conferred genotypes known to increase the risk of type 1 diabetes and a first-degree relative with type 1 diabetes [16]. In the TRIGR study, an extensively hydrolysed casein infant formula was compared with a regular formula based on cow’s milk. All participating infants were followed until the youngest child turned 10 years of age. Blood samples were collected at intervals of 3–12 months. The Divia ancillary study of the TRIGR cohort tests hypotheses related to the immune system, diet and virus infections in the development of type 1 diabetes. All children within the TRIGR study that developed islet autoimmunity (positivity for at least two out of four autoantibodies measured) were included as ‘cases’ in the TRIGR Divia ancillary study. For each case child (*n* = 244), two control children (*n* = 488) were randomly selected from children that did not develop positivity for two or more autoantibodies, matched for date of birth (±1 year) and country. The same matched control children were used also for those children that developed type 1 diabetes (*n* = 144). Written informed consent was collected from all families, signed by the legal guardian of the child. The study was approved by the ethics committees of all participating centres and was conducted according to the standards of the Declaration of Helsinki.

### Islet autoimmunity

Case children (*n* = 244) were defined as those with positivity, at any one sample, for two or more of the following autoantibodies: ICA, IAA, IA-2A and GADA. Autoantibodies were quantified with the use of specific radiobinding assays in the Scientific Laboratory, Children’s Hospital, University of Helsinki, Helsinki, Finland [16].

### HLA genotyping

HLA genotyping for the selected *DQB1* and *DQA1* alleles was performed using sequence-specific oligonucleotide hybridisation with the following genotypes regarded as eligible: (1) HLA DQB1*02/DQB1*03:02 (high risk); (2) HLA DQB1*03:02/x (*x* not DQB1*02, DQB1*03:01 or DQB1*06:02) (moderate risk); (3) HLA DQA1*05-DQB1*02/y (*y* not DQA1*02:01-DQB1*02, DQB1*03:01, DQB1*06:02 or DQB1*06:03) (mild risk); and (4) HLA DQA1*03-DQB1*02/y (*y* not DQA1*02:01-DQB1*02, DQB1*03:01, DQB1*06:02 or DQB1*06:03) (rare mild risk) [16].

### Serum 25OHD analyses

The serum samples were collected during 2002–2017. The samples were stored frozen at −70°C until analysed. Serum 25OHD concentration was determined by a chemiluminescent microparticle immunoassay by Architect *i* system (Abbott Laboratories, Abbott Park, IL, USA). The inter-assay CV of 25OHD was 4.3% and 3.4% at the levels of 35 nmol/l and 104 nmol/l, respectively. Bias compared to all-laboratory trimmed means in the Vitamin D International External Quality Assessment Scheme (DEQAS) was 6.4 ± 9.3% (mean ± SD), which is considered satisfactory. Furthermore, the laboratory participated in a standardisation study comparing the Architect method with LC–tandem MS [17], showing excellent agreement between the two methods at 25OHD concentrations relevant to this study. In this study, a serum 25OHD concentration of <30 nmol/l was considered as severe vitamin D deficiency.

### Statistical analyses

The average 25OHD concentration (nmol/l) across all age points was calculated for each participant. Separate averages for each participant were calculated based on the season of sample collection. Descriptive statistics based on these averages (means, SD and 95% CI) were calculated based on participant type (case or control), region, HLA type and season of sample collection and compared using general linear models. Conditional logistic regression models were used to assess the odds of islet autoimmunity for 1 nmol/l increase in 25OHD concentration. To account for possible confounding, we evaluated the associations between background variables and serum 25OHD concentrations. As expected, serum 25OHD concentrations were lower during the winter than during the summer. To adjust for this effect, month of sample collection was included in the conditional logistic regression models. All analyses were adjusted additionally for factors known to associate with the risk of type 1 diabetes (HLA genotype, maternal type 1 diabetes and sex). A *p* value <0.05 was considered statistically significant. The analyses were performed using SAS v9.4 (SAS, Cary, NC, USA). The TRIGR study is originally a clinical trial, but since no differences were detected between the treatment arm and the control arm [18], the original study setting did not require additional adjusting.

## Results

The distribution of the children according to the country or region and HLA genotype is shown in Table 1. Mean serum 25OHD concentration varied in different regions with children from Northern Europe (Finland and Sweden) having the lowest concentrations, and children from Southern Europe the highest concentrations (Table 2). Serum concentrations were higher in samples collected during the summer months than winter months (Table 2). No significant association was found between serum 25OHD concentration and birthweight (*p* = 0.89), mode of birth (*p* = 0.74), gestational age (*p* = 0.47), maternal age (*p* = 0.32) or maternal education (*p* = 0.35).

**Table 1:** Distribution of the case children (with positivity for at least two islet autoantibodies) and control children in relation to the country or region and HLA genotype

Characteristic	Case children *n* (%)	Control children *n* (%)
Country/region		
Northern Europe (Finland, Sweden)	60 (24.6)	120 (24.6)
Canada	68 (27.9)	136 (27.9)
USA	39 (16.0)	78 (16.0)
Australia	9 (3.7)	18 (3.7)
Central Europe I (Czech Republic, Estonia, Hungary, Poland)	38 (15.6)	76 (15.6)
Central Europe II (Germany, Luxembourg, Netherlands, Switzerland)	23 (9.4)	46 (9.4)
Southern Europe (Italy, Spain)	7 (2.9)	14 (2.9)
HLA genotype		
HLA-DQB1*0302/DQA1*05-DQB1*02	90 (36.9)	117 (24.0)
HLA-DQB1*03:02/x (*x* not DQB1*02, DQB1*03:01 or DQB1*06:02)	96 (39.3)	208 (42.6)
HLA-DQA1*05-DQB1*02/y (*y* not DQA1*02:01-DQB1*02, DQB1*03:01, DQB1*06:02 or DQB1*06:03) and HLA-DQA1*03-DQB1*02/y (*y* not DQA1*02:01-DQB1*02, DQB1*03:01, DQB1*06:02 or DQB1*06:03)	58 (23.8)	163 (33.4)

**Table 2:** Mean serum 25OHD concentration (nmol/l) across all age points in relation to the country or region, HLA genotype and season of sample collection

Characteristic	Case children (*N* = 244)		Control children (*N* = 488)	
	Mean (SD) 25OHD	95% CI	Mean (SD) 25OHD	95% CI
Overall	65.5 (22.4)	62.6, 68.3	67.2 (22.4)	65.2, 69.2
Country/region				
Northern Europe (Finland, Sweden)	54.0 (15.1)	50.1, 57.9	54.6 (18.5)	51.3, 58.0
Canada	69.4 (19.6)	64.6, 74.1	71.2 (17.5)	68.3, 74.2
USA	69.9 (23.7)	62.3, 77.6	67.9 (19.3)	63.6, 72.3
Australia	65.2 (14.0)	54.5, 76.0	73.1 (19.0)	63.6, 82.5
Central Europe I (Czech Republic, Estonia, Hungary, Poland)	69.0 (29.8)	59.2, 78.8	74.2 (30.5)	67.2, 81.2
Central Europe II (Germany, Luxembourg, Netherlands, Switzerland)	67.6 (24.2)	57.1, 78.1	71.8 (19.0)	66.2, 77.5
Southern Europe (Italy, Spain)	74.7 (24.1)	52.4, 97.0	70.8 (34.1)	51.2, 90.5
HLA genotype				
HLA-DQB1*03:02/DQA1*05-DQB1*02	67.8 (23.9)	62.8, 72.8	69.2 (24.9)	64.6, 73.8
HLA-DQB1*03:02/x (*x* not DQB1*02, DQB1*03:01 or DQB1*06:02)	63.4 (22.6)	58.8, 68.0	68.3 (22.3)	65.3, 71.4
HLA-DQA1*05-DQB1*02/y (*y* not DQA1*02:01-DQB1*02, DQB1*03:01, DQB1*06:02 or DQB1*06:03) and HLA-DQA1*03-DQB1*02/y (*y* not DQA1*0201-DQB1*02, DQB1*03:01, DQB1*06:02 or DQB1*06:03)	65.3 (19.6)	60.1, 70.4	64.3 (20.4)	61.2, 67.5
Season of sample collection				
Winter (November–May)	62.2 (25.0)	59.0, 65.4	64.1 (26.8)	61.7, 66.5
Summer (June–October)	72.8 (29.5)	68.7, 76.9	71.8 (29.1)	69.0, 74.6

The median age for the first seroconversion was 2.0 years (interquartile range 1.0–4.0). Of the case children (*n* = 244), 144 developed type 1 diabetes, the mean age of diagnosis being 6.0 years (interquartile range 2.9–9.4).

Among the children that developed islet autoimmunity, the mean serum 25OHD concentration was lower 18 months prior to the age of the first seroconversion in the case children (57.7 nmol/l) compared with that of the control children at the corresponding age (64.8 nmol/l) (*p* = 0.007) (Table 3). Among children that developed type 1 diabetes (*n* = 144), the mean serum 25OHD concentration was lower 18 months prior to the age of the first seroconversion (58.0 vs 65.0 nmol/l) (*p* = 0.018) than in their control counterparts at the corresponding age, and at 12 months of age (70.1 vs 75.9 nmol/l) (*p* = 0.031) (Tables 3, 4). All analyses were adjusted for month of sample collection, HLA genotype, maternal type 1 diabetes and sex.

**Table 3:** Mean serum 25OHD concentration (nmol/l) at 18, 12 and 6 months prior to the first seroconversion and at the first seroconversion

Time relative to first seroconversion	Case children^a^(*N* = 244)		Control children(*N* = 488)		OR (95% CI)^b^	*p* value^b^	Children with T1D(*N* = 144)		Control children(*N* = 288)		OR (95% CI) for children with T1D^b^	*p* value for children with T1D^b^
	Mean (SD)	*n*	Mean (SD)	*n*			Mean (SD)	*n*	Mean (SD)	*n*		
18 months before	57.7 (27.6)	84	64.8 (32.7)	177	0.980 (0.965, 0.994)	0.007	58.0 (27.9)	65	65.0 (33.9)	141	0.979 (0.961, 0.996)	0.018
12 months before	60.4 (29.4)	86	65.9 (33.2)	172	0.993 (0.980, 1.005)	0.24	60.5 (29.0)	72	65.7 (34.1)	145	0.992 (0.977, 1.007)	0.27
6 months before	72.4 (33.0)	100	74.1 (32.3)	200	0.999 (0.989, 1.008)	0.77	72.6 (33.4)	84	74.7 (33.4)	169	0.997 (0.987, 1.008)	0.63
At the first seroconversion	71.0 (26.6)	60	72.2 (27.2)	124	0.999 (0.982, 1.015)	0.86	71.3 (28.2)	51	72.1 (28.4)	104	0.997 (0.979, 1.016)	0.79

^a^Children with positivity for at least two islet autoantibodies

^b^Adjusted for month of sample collection, HLA genotype, maternal type 1 diabetes and sex

T1D, type 1 diabetes

**Table 4:** Mean serum 25OHD concentration (nmol/l) according to the calendar age

Calendar age	Case children^a^(*N* = 244)		Control children(*N* = 488)		OR (95% CI)^b^	*p* value^b^	Children with T1D(*N* = 144)		Control children(*N* = 288)		OR (95% CI) for children with T1D^b^	*p* value for children with T1D^b^
	Mean (SD)	*n*	Mean (SD)	*n*			Mean (SD)	*n*	Mean (SD)	*n*		
Average (across all visits)	65.5 (22.4)	244	67.2 (22.4)	488	0.990 (0.975, 1.005)	0.20	63.7 (21.8)	144	67.4 (23.7)	288	0.980 (0.950, 1.011)	0.20
0 months	40.1 (21.4)	210	41.9 (25.7)	428	0.994 (0.985, 1.004)	0.22	38.8 (20.9)	121	42.9 (28.4)	254	0.992 (0.978, 1.006)	0.27
6 months	75.3 (39.3)	223	78.8 (38.1)	463	0.996 (0.990, 1.001)	0.10	74.9 (36.9)	129	79.5 (40.0)	273	0.994 (0.987, 1.001)	0.11
12 months	74.1 (28.2)	233	76.0 (28.6)	453	0.997 (0.990, 1.004)	0.36	70.1 (26.2)	135	75.9 (29.3)	265	0.989 (0.978, 0.999)	0.031
18 months	69.0 (25.2)	193	69.3 (24.8)	399	0.997 (0.989, 1.006)	0.53	67.0 (25.4)	102	69.0 (24.5)	223	0.989 (0.976, 1.003)	0.12

^a^Children with positivity for at least two islet autoantibodies

^b^Adjusted for month of sample collection, HLA genotype, maternal type 1 diabetes and sex

T1D, type 1 diabetes

Only 3% of the case children and 2% of the control children had severe vitamin D deficiency at any time point (serum 25OHD concentration <30 nmol/l) (*p* = 0.27).

## Discussion

In this study, we investigated the association between serum 25OHD concentration during early childhood and the development of islet autoimmunity and type 1 diabetes. Vitamin D status was determined using two time scales: time in relation to the age at the initial seroconversion and to the calendar age. We found that the mean serum 25OHD concentration was lower 18 months prior to the initial seroconversion in the case children compared with their control children. In addition, children who progressed to clinical type 1 diabetes had a lower serum 25OHD concentration 18 months prior to the initial seroconversion and at 12 months of age compared with control children. All other time points showed similar vitamin D status in case and control children.

The strengths of the present study include a unique sample set. The measurement of serum 25OHD concentration from multiple time points as well as from multiple study locations together with information on the HLA-conferred genetic risk allowed us a more detailed analysis of vitamin D status as a possible determinant of the risk for islet autoimmunity and type 1 diabetes. In addition, the fact that we only included those with two or more diabetes-associated autoantibodies confirms the reliability of the islet autoimmunity status. Most importantly, in addition to the calendar age, we were able to analyse the association between the disease process of type 1 diabetes and vitamin D status in relation to the age at initial seroconversion. The inclusion of multiple time points prior to the first seroconversion provided information on the importance of timing of inadequate vitamin D supply.

Our results do not support a strong role for vitamin D in the disease process leading to type 1 diabetes. This is consistent with results from the DIPP and DAISY birth cohort studies [2–4]. Recent findings suggest that vitamin D deficiency may be a risk factor for those carrying certain genotypes of the *VDR* gene [5,13]. A limitation of the present study is the lack of genetic data to assess whether the differences that were detected would be stronger when analysed according to the genetic factors that affect vitamin D metabolism. Also, due to inadequate statistical power, we were not able to analyse the association separately according to country, region or ethnicity. A further challenge is the fact that participants in the TRIGR study represent a specific high-risk population in terms of type 1 diabetes, since the inclusion criteria included both a genetic HLA-conferred risk and a first-degree relative with type 1 diabetes. The TRIGR, the TEDDY and the DIPP studies all have distinct study populations, inclusion criteria and definition of islet autoimmunity. Therefore, the results cannot be directly compared or generalised to other populations. For instance, it should be noted that results may be different in vitamin D-deficient populations.

Our results indicate that if vitamin D status modifies the risk of islet autoimmunity or type 1 diabetes in genetically susceptible children, the possible effect may relate to the early stage of the disease process (i.e. before seroconversion). It has to be noted, though, that within this study setting, we were not able to identify the possible effect of vitamin D at later stages of the disease. The fact that half of the case children developed islet autoimmunity at less than 2 years of age means they were not all included when comparing vitamin D status between case and control children 18 months before the first seroconversion. Since a difference in vitamin D status between the case and control children was identified 18 months before the first seroconversion, this may imply that the possible effect is stronger in those children who developed islet autoimmunity at a slightly older age.

The proposed mechanisms of the possible association between vitamin D and type 1 diabetes are related to the various effects of vitamin D on the immune system. The importance of vitamin D in the immune system is demonstrated by the presence of VDRs in a majority of the cells of the immune system [19]. Immune cells also express vitamin D-activating enzymes that locally convert inactive vitamin D to its active form [19]. Vitamin D may also interfere with gut microbiota [20,21] that has also been associated with the early stages of the disease process of type 1 diabetes [22], highlighting the complexity of the possible association between vitamin D and type 1 diabetes. Given that a clear difference in vitamin D status was seen in the present study before seroconversion and that the children developed islet autoimmunity at a very young age (at a median age of 2 years), the possible developmental origin and therefore the role of prenatal vitamin D status may be of interest. However, several previous studies have failed to show an association between 25OHD concentration during pregnancy or at birth with the risk of type 1 diabetes in the child [8,9].

In the present study, vitamin D deficiency was rare. It has been suggested that rather than being a cause of any disease, poor vitamin D status may be a consequence of ill health [23]. In Finland, where vitamin D deficiency has been prevalent until recent years, it was noticed that an increase in mean serum 25OHD concentrations as a consequence of vitamin D fortification, preceded a plateau in the increase in type 1 diabetes incidence [24]. It is not possible to evaluate any causality in this temporal association.

The results suggest that early postnatal vitamin D may confer protection against the development of type 1 diabetes.

## Electronic supplementary material

ESM List of TRIGR Investigators (PDF 311 kb)

## Data Availability

The authors confirm that, for approved reasons, some access restrictions apply to the datasets generated during and/or analysed during the current study underlying the findings. Researchers interested in using the data are required to follow the terms of a number of clauses designed to ensure the protection of privacy and compliance with relevant regulation. Data are available upon request due to ethical restrictions, pending approval from the relevant ethical committees.

## Abbreviations

DAISY: Diabetes Auto Immunity Study in the Young
DIPP: Diabetes Prediction and Prevention
GADA: GAD autoantibody
IAA: Insulin autoantibody
IA-2A: Insulinoma-associated antigen-2 antibody
ICA: Islet cell autoantibody
25OHD: 25-Hydroxyvitamin D
TEDDY: Environmental Determinants of Diabetes in the Young
TRIGR: Trial to Reduce IDDM in the Genetically at Risk
VDR: Vitamin D receptor
ZnT8A: Zinc transporter-8 autoantibody
