# Supplementary material for: Serum 25-hydroxyvitamin D concentration in childhood and risk of islet autoimmunity and type 1 diabetes: the TRIGR nested case–control ancillary study
Source: Diabetologia. 2020 Jan 7;63(4):780–7. doi: 10.1007/s00125-019-05077-4 (PMC7054378; doi:10.1007/s00125-019-05077-4)
Supplement: Supplementary file 1 — (PDF 311 kb) [file 125_2019_5077_MOESM1_ESM.pdf]

LIST OF TRIGR INVESTIGATORS FOR PUBLICATIONS / version December 2019

Administration/ Country	Study center	Last name	First name	Position
**Data Safety Monitoring Board**		Mandrup-Poulsen,	Thomas	Chair University of Copenhagen, Copenhagen, Denmark
		Arjas	Elias	Member, University of Helsinki, Helsinki, Finland
		Läärä	Esa	Member, Unioversity of Oulu, Oulu, Finland
		Lernmark	Åke	Member, University of Lund, Malmö, Sweden
		Schmidt	Barbara	Member, University of Pennsylvania, Philadelphia, PA, USA
		Krischer	Jeffrey P.	Observer, University of South Florida. Tampa FL, USA
**International Coordinating Center (ICC), University of Helsinki, Helsinki, Finland**		Åkerblom	Hans K. † *	PI of the Study until 30.6.08, Deputy PI from 1.7.2008
		Hyytinen	Mila	European Study Monitor
		Knip	Mikael	Deputy PI until 30.6.2008, PI of the Study from 1.7.2008, National Investigator
		Koski	Katriina	European Study Monitor
		Koski	Matti	IT Specialist
		Pajakkala	Eeva	European Study Monitor
		Salonen	Marja	Study Coordinator
**Data Management Unit (DMU), University of South Florida, Tampa, FL, USA**		Cuthbertson	David	Biostatistician
		Krischer	Jeffrey P.	PI of the DMU
		Shanker	Linda	Coordinator

LIST OF TRIGR INVESTIGATORS FOR PUBLICATIONS / version December 2019

Administration/ Country	Study center	Last name	First name	Position
**Canadian Coordinating Center, University of Western Ontario, London, and University of Ottawa, ON**		Bradley	Brenda	National Coordinator
		Dosch	Hans-Michael	Co-Investigator for Canada
		Dupré	John	Co-PI for North America and National Investigator until 08/12/2015, Executive Committee
		Fraser	William	Co-Investigator for Canada Executive Committee
		Lawson	Margaret	Co-Investigator for Canada Executive Committee
		Mahon	Jeffrey L.	Co-PI for North America and National Investigator after 08/12/2015, Executive Committee
		Sermer	Mathew	Co-Investigator for Canada, Executive Committee
		Taback	Shayne P.	Co-Investigator for Canada, Executive Committee
**USA Coordinating Center, University of Pittsburgh, PA and University of Washington, Seattle, WA**		Becker	Dorothy	Co-PI for North America, National Investigator, Executive Committee
		Franciscus	Margaret	National Coordinator
		Nucci	Anita	National Coordinator, Nutrition Coordinator of North America
		Palmer	Jerry	Executive Committee

LIST OF TRIGR INVESTIGATORS FOR PUBLICATIONS / version December 2019

Administration/ Country	Study center	Last name	First name	Position
**Nutritional Epidemiology Unit, National Institute for Helth and Welfare, Helsinki, Finland**		Virtanen	Suvi M.	Head of Nutritional Epidemiology Unit
**Australia**	**AUS01 - Westmead - Children’s Hospital**	Catteau	Jacki	National Coordinator
		Howard	Neville	National Investigator
	**AUS02 - Newcastle - John Hunter Children’s Hospital**	Crock	Patricia	Local Investigator
	**AUS03 - Sydney - Sydney Children’s Hospital**	Craig	Maria	Local Investigator
**Canada**	**CAN01 - London - St. Joseph's Health Care Centre**	Clarson	Cheril L.	Local Investigator
		Bere	Lynda	Coordinator
	**CAN02 - Vancouver - Children's and Women's Health Centre of British Columbia**	Thompson	David	Local Investigator
		Metzger	Daniel	Local Investigator
		Marshall	Colleen	Coordinator (In Transition)
		Kwan	Jennifer	Coordinator (In Transition)
	**CAN03 - Calgary - Alberta Children's Hospital**	Stephure	David K.	Local Investigator
		Pacaud	Daniele	Co-Investigator
		Schwarz	Wendy	Coordinator

LIST OF TRIGR INVESTIGATORS FOR PUBLICATIONS / version December 2019

Administration/ Country	Study center	Last name	First name	Position
	**CAN04 - Edmonton - Walter MacKenzie Health Sciences**	Girgis<br>Thompson	Rose<br>Marilyn	Local Investigator<br>Coordinator
	**CAN05 - Winnipeg - Health Sciences Centre**	Taback<br>Catte	Shayne P<br>Daniel	Local Investigator<br>Coordinator
	**CAN06 - Ottawa - Children's Hospital of Eastern Ontario and The Ottawa Hospital**	Lawson<br>Bradley	Margaret L<br>Brenda	Local Investigator<br>Coordinator
	**CAN07 - Toronto Mount Sinai Hospital/Hospital for Sick Children**	Daneman<br>Sermer<br>Martin	Denis<br>Mathew<br>Mary-Jean	Local Investigator<br>Co-Investigator<br>Coordinator
	**CAN08 - Quebec - CHUQ**	Morin<br>Frenette<br>Ferland	Valérie<br>Line<br>Suzanne	Local Investigator<br>Co-Investigator<br>Coordinator
	**CAN09 - Saint John – Regional Hospital**	Sanderson<br>Heath	Susan<br>Kathy	Local Investigator<br>Coordinator
	**CAN10 - Montreal - L' Hôpital Sainte-Justine**	Huot<br>Gonthier<br>Thibeault	Céline<br>Monique<br>Maryse	Local Investigator<br>Co-Investigator<br>Coordinator
	**CAN11 - Montreal Children's Hospital**	Legault<br>Laforte	Laurent<br>Diane	Local Investigator<br>Coordinator

LIST OF TRIGR INVESTIGATORS FOR PUBLICATIONS / version December 2019

Administration/ Country	Study center	Last name	First name	Position
	**CAN12 - Halifax - IWK Health Centre/Dalhousie**	Cummings<br>Scott	Elizabeth A<br>Karen	Local Investigator<br>Coordinator
	**CAN13 - St. John's - Janeway Child Health Center**	Bridger<br>Crummell	Tracey<br>Cheryl	Local Investigator<br>Coordinator
	**CAN14 - Kingston - Kingston General Hospital/ Queen's University**	Houlden<br>Breen	Robyn<br>Adriana	Local Investigator<br>Coordinator
	**CAN15 - Regina - Regina Qu'Appelle**	Carson<br>Kelly	George<br>Sheila	Local Investigator<br>Coordinator
	**CAN16 - Saskatoon - Royal University Hospital**	Sankaran<br>Penner	Koravangattu<br>Marie	Local Investigator<br>Coordinator
	**CAN17 - Peterborough - Peterborough Regional Health Centre**	White<br>King	Richard A<br>Nancy	Local Investigator<br>Coordinator
	**CAN18 - Victoria - Vancouver Island Health Research Centre**	Popkin<br>Robson	James<br>Laurie	Local Investigator<br>Coordinator
**Czech Republic**	**CZE01 - Prague - Faculty Hospital Kralovske Vinohrady**	Al Taji<br>Mendlova<br>Romanova<br>Vavrinec	Eva<br>Pavla<br>Martina<br>Jan † *	National Investigator/Coordinator<br>National Coordinator<br>Co-Investigator<br>National Investigator

LIST OF TRIGR INVESTIGATORS FOR PUBLICATIONS / version December 2019

Administration/ Country	Study center	Last name	First name	Position
		Vosahlo	Jan	Co-Investigator
	**CZE02 - Brno - Hospital Milosrdnych Bratri**	Brazdova	Ludmila	Local Investigator
	**CZE03- Olomouc - Faculty Hospital Olomouc**	Venhacova	Jitrenka	Local Investigator
		Venhacova	Petra	Co-Investigator
	**CZE04 - Usti nad Labem - Hospital of Masryk**	Cipra	Adam	Local Investigator
	**CZE05 - Ceske Budejovice - Hospital Ceske Budejovice**	Tomsikova	Zdenka	Local Investigator
	**CZE06 - Plzen - Faculty Hospital Plzen**	Paterová	Petra	Local Investigator
	**CZE07 - Zlin - Hospital of Bata**	Gogelova	Pavla	Local Investigator
**Estonia**	**EST01 - Tallinn - Tallinn Children's Hospital**	Einberg	Ülle	Co-Investigator
		Riikjärv	Mall-Anne	Local Investigator
	**EST02 - Tartu - Tartu University Children's Hospital**	Ormisson	Anne	National Investigator
		Tillmann	Vallo	Co-Investigator

LIST OF TRIGR INVESTIGATORS FOR PUBLICATIONS / version December 2019

Administration/ Country	Study center	Last name	First name	Position
**Finland**	**FIN01 - Helsinki – Children's Hospital, University of Helsinki**	Johansson	Susanne	National Coordinator
		Kleemola	Päivi	National Coordinator
		Parkkola	Anna	Local Investigator
	**FIN02 - Helsinki - Department of Obstetrics and Gynecology, University of Helsinki**	Järvenpää	Anna-Liisa	Local Investigator
	**FIN03 - Espoo - Jorvi Hospital**	Hämäläinen	Anu-Maaria	Local Investigator
		Kiiveri	Sanne	Local Investigator
	**FIN04 - Kotka - Kymenlaakso Central Hospital**	Salonen	Maria	Local Investigator
		Tenhola	Sirpa	Local Investigator
	**FIN05 - Lahti - Paijat-Hame Central Hospital**	Salonen	Pia	Local Investigator
	**FIN06 - Tampere - Department of Pediatrics, Tampere University Hospital**	Jason	Eeva	Local Investigator
		Selvenius	Jenni	Local Investigator
		Siljander	Heli	Co-Investigator
	**FIN07 - Pori - Satakunta Central Hospital**	Ylitalo	Samuli	Local Investigator
	**FIN08 - Jyväskylä - Central Finland Central Hospital**	Paajanen	Ilkka	Local Investigator

LIST OF TRIGR INVESTIGATORS FOR PUBLICATIONS / version December 2019

Administration/ Country	Study center	Last name	First name	Position
	**FIN09 - Seinäjoki - South Ostrobotnia Central Hospital**	Talvitie	Timo	Local Investigator
	**FIN10 - Hyvinkää - Hyvinkää Hospital**	Lindström	Kaija	Local Investigator
	**FIN11 - Kuopio - Department of Pediatrics, Kuopio University Hospital**	Huopio<br>Pesola	Hanna<br>Jouni	Local investigator<br>Co-Investigator
	**FIN12 - Oulu - Department of Pediatrics, Oulu University Hospital**	Veijola<br>Tapanainen	Riitta<br>Päivi	Local Investigator<br>Co-Investigator
	**FIN13 - Hämeenlinna - Kanta-Hame Central Hospital**	Alar	Abram	Local Investigator
	**FIN14 - Vaasa - Vaasa Central Hospital**	Popov	Erik	Local Investigator
	**FIN15 - Lappeenranta - South Carelian Central Hospital**	Virransalo	Ritva	Local Investigator
	**FIN16 - Mikkeli - Mikkeli Central Hospital**	Nykänen	Päivi	Local Investigator

LIST OF TRIGR INVESTIGATORS FOR PUBLICATIONS / version December 2019

Administration/ Country	Study center	Last name	First name	Position
**Germany**	**GER01 - Hannover - Kinder- und Jugendkrankenhaus – Auf der Bult**	Aschemeier	Bärbel	National Coordinator
		Danne	Thomas	National Investigator
		Kordonouri	Olga	Co-Investigator
**Hungary**	**HUN01 - Budapest - Semmelweis Medical University**	Krikovszky	Dóra	Co-Investigator
		Madácsy	László	National Investigator
**Italy**	**ITA01 - Rome - University Campus Bio-Medico of Rome**	Khazrai	Yeganeh Manon	Local Coordinator
		Maddaloni	Ernesto	Local Coordinator
		Pozzilli	Paolo	National Investigator
	**SAR01 - Cagliari - St. Michele Hospital**	Mannu	Carla	Local Coordinator
		Songini	Marco	National Investigator
**Luxembourg**	**LUX01 - Luxembourg - Centre Hospitalier de Luxembourg**	de Beaufort	Carine	National Investigator
		Schierloh	Ulrike	Co-Investigator
**The Netherlands**	**NET01 - Rotterdam - Sophia Children's Hospital**	Bruining	Jan † *	National Investigator
		Bisschoff	Margriet	National Coordinator
**Poland**	**POL01 - Wroclaw - Medical University of Wroclaw**	Basiak	Aleksander	Co-Investigator
		Wasikowa	Renata	National Investigator
	**POL02 - Krakow - Polish-American Children's Hospital**	Ciechanowska	Marta	Local Investigator
		Deja	Grazyna	Co-Investigator

LIST OF TRIGR INVESTIGATORS FOR PUBLICATIONS / version December 2019

Administration/ Country	Study center	Last name	First name	Position
	**POL03 - Katowice - Medical University of Silesia**	Jarosz-Chobot	Przemyslawa	Local Investigator
	**POL04 - Lodz - Medical University of Lodz**	Szadkowska	Agnieszka	Co-Investigator
	**POL05 - Lodz - Polish Mother's Memorial Hospital (I.C.Z.M.P)**	Cypryk<br>Zawodniak-Szalapska	Katarzyna<br>Malgorzata	Local Investigator<br>Co-Investigator
**Spain**	**SPA01 - Cruces University Hospital-UPV/EHU-CIBERDEM/CIBERER, Barakaldo, Spain**	Castano<br>Chueca<br>Gonzalez Frutos	Luis<br>Maria<br>Teba	National Investigator<br>Co-Investigator<br>Local Coordinator
	**SPA02 - Madrid - Hospital Clinico San Carlos**	Serrano-Ríos<br>Martínez-Larrad<br>Hawkins	Manuel<br>María Teresa<br>Federico Gustavo	National Investigator<br>Local Coordinator<br>Co-Investigator
	**SPA03 - Madrid - Hospital Gregorio Marañon**	Rodriguez Arnau	Dolores	Co-Investigator
**Sweden**	**SWE01 - Linköping - University of Linköping**	Ludvigsson<br>Smolinska Konefal	Johnny<br>Malgorzata	National Investigator<br>National Coordinator
	**SWE02 - Uddevalla - Uddevalla Hospital**	Hanas	Ragnar	Local Investigator
	**SWE03 - Göteborg - GothenburgThe Queen**	Lindblad	Bengt	Local Investigator

LIST OF TRIGR INVESTIGATORS FOR PUBLICATIONS / version December 2019

Administration/ Country	Study center	Last name	First name	Position
	**Silvia Children's Hospital**			
	**SWE05 - Halmstad - Halmstad Hospital**	Nilsson	Nils-Östen	Local Investigator
	**SWE06 - Trollhättan - Trollhättan Hospital**	Fors	Hans	Local Investigator
	**SWE07 - Norrköping - Vrinnevi Hospital**	Nordwall	Maria	Local Investigator
	**SWE08 - Borås - Borås Hospital**	Lindh	Agne	Local Investigator
	**SWE09 - Karskrona - Karlskrona Hospital**	Edenwall	Hans	Local Investigator
	**SWE10 - Örebro - University Hospital**	Åman	Jan	Local Investigator
	**SWE11 - Jönköping - Ryhovs Hospital**	Johansson	Calle	Local Investigator
**Switzerland**	**SWT01 - Zürich - University Children's Hospital**	Gadient<br>Konrad<br>Schoenle	Margrit<br>Daniel<br>Eugen	Local Coordinator<br>National Investigator<br>National Investigator
**USA**	**USA01 - Pittsburgh - Children's Hospital of Pittsburgh**	Becker	Dorothy	USA National Investigator / Pittsburgh Local Investigator
		Daftary	Ashi	Co-Investigator
		Klein	Mary Beth	Pittsburgh Coordinator

LIST OF TRIGR INVESTIGATORS FOR PUBLICATIONS / version December 2019

Administration/ Country	Study center	Last name	First name	Position
		Gilmour	Carol	Co-Investigator
	**USA02 - Seattle - VA Puget Sound Health Care System and University of Washington**	Palmer	Jerry	Local Investigator
		Palmer	Patty	Local Investigator
		Malone	Patty	Coordinator
	**USA03 - St. Louis - Washington University**	Tanner-Blasiar	Marilyn	Coordinator
		White	Neil	Local Investigator
	**USA04 - Los Angeles - Mattel Children's Hospital of UCLA**	Devaskar	Uday	Local Investigator
		Horowitz	Heather	Coordinator/dietitian
		Rogers	Lisa	Coordinator/dietitian
	**USA05 - Ponce - Ponce School of Medicine**	Colon	Roxana	Coordinator
		Frazer	Teresa	Co-Investigator
		Torres	Jose	Local Investigator
	**USA06 - New York - Naomie Berrie Diabetes Center**	Goland	Robin	Local Investigator
		Greenberg	Ellen	Coordinator
		Schachner	Holly	Co-Investigator
		Softness	Barney	Co-Investigator
**Laboratories**	**HLA-typing Laboratory – University of Turku – Finland**	Ilonen	Jorma	Head of HLA-typing Laboratory
	**HLA-typing Laboratory – University of Pittsburgh - PA - USA**	Trucco	Massimo	Head of HLA-typing Laboratory
		Nichol	Lynn	Chief Technician

LIST OF TRIGR INVESTIGATORS FOR PUBLICATIONS / version December 2019

Administration/ Country	Study center	Last name	First name	Position
	**Cow's Milk Antibody Laboratory – University of Helsinki – Finland**	Savilahti	Erkki	Head of Cow's Milk Antibody Laboratory
	**Autoantibody Laboratory – University of Helsinki – Finland**	Härkönen	Taina	Co-Investigator
		Knip	Mikael	Head of Antibody Laboratory
	**T-Cell Laboratory - Helsinki – Finland**	Vaarala	Outi	Head of T-cell Laboratory
		Luopajärvi	Kristiina	Co-Investigator
	**T-Cell Laboratory – Hospital for Sick Children, Toronto - ON - Canada**	Dosch	Hans-Michael	Head of T-Cell Laboratory
